# Reduced lung function among non-smoking workers in the paper industry in Ethiopia: a comparative cross-sectional study

**DOI:** 10.1007/s00420-025-02163-6

**Published:** 2025-08-26

**Authors:** Ararso Tafese, Bente E. Moen, Abera Kumie, Samson Wakuma Abaya, Wakgari Deressa, Teferi Abegaz, Magne Bråtveit

**Affiliations:** 1 Department of Preventive Medicine, School of Public Health, Addis Ababa University, P.O. Box 90861000, Addis Ababa, Ethiopia; 2 Department of Global Public Health and Primary Care, Centre for International Health, University of Bergen, 5009 Bergen, Norway; 3 Department of Global Public Health and Primary Care, University of Bergen, 5009 Bergen, Norway

**Keywords:** Lung function, Cumulative dust exposure, Paper dust, Occupational exposure, Paper industry

## Abstract

**Purpose:**

Exposure to paper dust in the workplace might increase the risk of reduced lung function. This study aims to evaluate the relationship between paper dust exposure and lung function among workers in the paper industry in Ethiopia.

**Methods:**

A comparative cross-sectional study assessed lung function in workers exposed to dust in the paper industry and compared them with controls from the water bottling industry. Lung function tests were conducted using a portable spirometer. A job exposure matrix was developed to estimate cumulative exposure to inhalable paper dust. Analysis of covariance was performed to compare mean lung function between exposed and control groups and multivariate linear regression analysis was carried out for workers exposed to paper dust.

**Results:**

There was a significant difference in forced expiratory volume in one second (FEV_1_) between the groups. FEV_1_ was 2.62 l in the exposed group, compared to 2.97 l in the control group. The multiple linear regression analysis revealed that cumulative paper dust exposure was associated with a reduction in both FEV_1_ and forced vital capacity (FVC). Each unit increases in dust exposure (measured in mg year/m^2^) was associated with a 0.010 l decrease in both FEV_1_ and FVC in females and a 0.005-liter decrease in males.

**Conclusions:**

The findings indicate a significant exposure-response relationship between cumulative paper dust exposure and a decline in lung function with the effect being more pronounced among female workers than among males. Based on these results, we recommend the paper industry to implement effective dust control strategies.

## Background

Workplace exposures significantly contribute to the burden of occupational respiratory disease, with important implications for clinical research and policy development (Blanc et al. 2019) A systematic review pointed out that occupational dust exposure is one of the main causes of chronic obstructive pulmonary disease (COPD) (Oxman et al. 2012). While smoking is the leading risk factor for chronic respiratory disease among men, ambient particulate matter represents the primary risk factor among women (GBD 2020). Lung function measurement at the workplace is the most important diagnostic tool for the early and accurate detection of pulmonary dysfunction in workers, and it plays a key role in establishing exposure-response relationships (Crapo 1994).

Several studies have shown associations between exposure to paper dust and respiratory impairment among workers in the soft tissue industry, where they produce items such as napkins, facial tissues, towels, and sanitary napkins (Andersson et al. 2020; Ericsson et al. 1988; Järvholm et al. 1988; Kraus et al. 2002, 2004; Thorén et al. 1989). Similarly, employees in the paper manufacturing sector experience respiratory impairments due to paper dust exposure while producing a variety of products, including white bond paper, test liners, kraft liners, ream wrappers, paper tubes, and corrugated cartons (Negash et al. 2023; Tafese et al. 2024a; Toren et al. 1996; Torén et al. 1994; Zuskin et al. 1998). Some of these investigations yielded inconclusive results due to the utilization of various dust sampling methods, such as personal total dust sampling, with reported arithmetic mean (AM) exposure levels ranging from 2 to 40 mg/m^3^. The AM of personal inhalable paper dust exposure ranged from 4.5 to 12.4 mg/m^3^ (Kraus et al. 2004; Tafese et al. 2024a), while respirable paper dust ranged from 0.28 to 2.16 mg/m^3^ (Kraus et al. 2004; Zuskin et al. 1998). Some of these studies did not incorporate estimations of cumulative dust exposure (Järvholm et al. 1988; Negash et al. 2023; Thorén et al. 1989). Despite these limitations, studies have indicated that exposure to paper dust is associated with a decline in lung function (Andersson et al. 2020; Ericsson et al. 1988; Kraus et al. 2004; Zuskin et al. 1998), although one study did not find a significant association (Thorén et al. 1989).

No studies have investigated lung function among workers in the Ethiopian paper industry. Most previous research has focused on the soft paper sector in European countries. However, recent studies have reported an arithmetic mean of 4.5 mg/m^3^ for personal inhalable paper dust levels in Ethiopian paper factories (Tafese et al. 2024a), and found that cumulative paper dust exposure was associated with chronic cough (Tafese et al. 2024b). These findings indicate that paper industry workers might also develop reduced lung function. Ethiopia imports substantial quantities of paper and paper products to meet domestic demand. However, the Ethiopian government has recently increased its efforts to stimulate local paper production in order to better address the growing need for paper and paper products. Therefore, we aimed to assess lung function among paper industry workers in Ethiopia compared to controls, and to examine the exposure-response relationship between cumulative paper dust exposure and lung function among the paper industry workers.

## Materials and methods

### Study population and design

This study was conducted in four paper manufacturing industries in Ethiopia, utilizing a comparative cross-sectional design, from March 2023 to May 2023. We selected the industries based on their size (large-scale industries) and the type of raw materials used. The same four paper mills were included in our previous studies on dust exposure and respiratory symptoms (Tafese et al. 2024a, b). Each of the four paper mills employed between 256 and 559 workers, qualifying them as large-scale industries according to the Eurostat definition (Eurostat 2010), which classifies businesses with more than 250 employees as large-scale.

### Description of the industries and the paper-making process

The four mills are described in detail in Tafese et al. (2024a). In brief, all primarily utilized recycled paper as raw material. Two of the mills also used imported pulp in addition to recycled paper, while the other two relied exclusively on recycled paper to produce paper and paper products. Their daily production capacity ranged from 30 to 100 tons.

The paper-making process combines manual labor and mechanized systems, taking place in designated work areas with no control rooms present in any mills. Preparation begins with selecting and sorting raw materials, mainly recycled paper and pulp, which are pulped to break down fibers and remove contaminants. The pulp may be bleached for brightness. The pulp slurry is then placed on a moving wire mesh conveyor, where water drains away, and fibers bond to form a continuous wet sheet. This sheet passes through press rollers to remove moisture and is dried with heated rollers. A sizing treatment may enhance water and ink resistance. In the finishing stage, the dried paper is cut, trimmed, and may be coated or converted into products like packaging materials. Finally, the products undergo quality inspection and are packaged for transport and storage. All four industries were involved in similar production of a wide variety of paper and paper products. This included high-quality white bond paper commonly used for printing, as well as fluting paper, kraft liner, test liner, paper tubes, and cones (Tafese et al. 2024a). They also produced cardboard and corrugated carton boxes, which are essential for packaging and transporting products.

A control group was selected from the water-bottling industry, assuming minimal dust exposure, as previous research in a similar study area reported a mean dust exposure level of 0.33 mg/m^2^ in water-bottling facilities (Abaya et al. 2018).

### Sample size determination and selection procedures

To determine the sample size, we used the mean difference with a 2:1 ratio for exposed and control groups, based on data from a previous study in Germany’s soft tissue paper industry (Kraus et al. 2004), which focused on forced expiratory volume in one second (FEV_1_) as the primary outcome. The sample size was calculated using the Openepi (http://www.openepi.com/SampleSize/SSMean.htm), incorporating the mean and standard deviation for FEV_1_ (exposed group: mean = 101% ± 18.1, control group: 107.3% ± 15.8), with a significance level of 0.05 and a power of 90%. Finally, we added 10% to account for non-response rate, resulting in 363 participants: 242 from the exposed group (paper industry workers) were randomly selected from 1405 workers and 121 from the control group (water bottling workers) were also randomly selected from 720 workers to participate in the study. One worker who had previously undergone abdominal surgery and two pregnant female workers were excluded during the data collection period.

### Lung function measurements

Lung function tests were carried out per the guidelines for a spirometer (ATS 1995). We used a portable computer-based spirometer SPIROBANK II BASIC-Handheld (MIR 2023). The participants’ standing height and weight were measured using a standard weighing scale. The tests were performed during the day shift work, between 08:00 and 16:00, with the participants in a sitting position. Before conducting the test, we held a brief meeting with employees and supervisors to discuss the purpose of the study. During this meeting, we instructed the employees to refrain from alcohol consumption for at least 4 h, avoid strenuous physical activity within 30 min prior to the test, wear loose-fitting clothing, and avoid consuming a large meal within 2 h prior before the test.

We retained three acceptable manoeuvres with consistent (repeatable) results and selected the best of these for analysis based on the ATS criteria for acceptability: at least 3 good efforts—quick start, no coughing, no early termination (not less than 6 s), smooth and continuous exhalation. Repeatability was assessed by the best two FEV_1_ and FVC values, which should be within 150 mL of each other. Since there are currently no reference equations for the Ethiopian population to calculate predicted values, only the absolute mean values for lung function were provided in the results. The lung function parameters forced vital capacity (FVC) and forced expiratory volume in one second (FEV_1_), along with the ratio of FEV_1_/FVC, were registered. Participants with FEV_1_/FVC < 0.70 were classified as having airflow limitation (Vogelmeier et al. 2017). We excluded 83 tests among workers exposed to paper dust and 27 from water bottling workers due to invalid spirometry readings.

Participants’ height and weight were measured during the assessment while they were dressed in light clothing and without shoes. Weight was recorded in kilograms using a standard weighing scale, while height was measured in meters with participants standing upright, feet together, eyes level, and looking straight ahead. The weighing scales were calibrated using the automatic zero function, which resets the display to zero upon activation. Body mass index (BMI) was calculated for each participant using the formula weight (kg)/height^2^ (m^2^).

Alongside the spirometry tests, data was collected from each worker on socio-demographic factors including age, gender, educational background, and work history. This included weekly working hours (in hours), years of employment in the paper industry, previous experience in other dusty sectors (yes or no), departments and tenure in each category (preparation, paper machine, finishing, and packing), personal history of respiratory illnesses (yes or no), use of biofuels for cooking at home (yes or no), and smoking status (current or former smoker). The initial data was recorded in English and later translated into Afan Oromo and Amharic for fieldwork data collection.

### Exposure assessment

In a previous study (Tafese et al. 2024a), we collected 150 personal inhalable paper dust samples from four distinct paper industries, each with four departments, resulting in an overall AM paper dust exposure of 4.5 mg/m^3^. Specifically, we collected ten personal inhalable paper dust samples from each of the 16 departments using the Institute of Occupational Medicine (IOM) samplers head made by SKC Ltd, running at a flow rate of 2.0 l min^−1^ (Skaugset et al. 2013). We developed a Job Exposure Matrix (JEM) (Neitzel et al. 2020; Tafese et al. 2024b) using the mean personal inhalable paper dust exposure data from each department: preparation, paper machine, finishing, and packing, across the four industries. Most of the workers had experience in multiple departments during the study period. For each paper worker, we calculated the cumulative dust exposure as the sum of the products of the AM of inhalable paper dust (mg/m^3^) in each department within the specific factory and the number of years the individual was employed in that department. The AM cumulative paper dust exposure was 30.3 mg year/m^3^, ranging from 2.5 to 134.1 mg year/m^3^.

### Data management and analysis

Data was analysed using SPSS version 26 (IBM Corp., Armonk, NY, USA). Covariance (ANCOVA) analysis was performed to compare mean lung function parameters between the paper industry workers and control groups, adjusting for age, sex and height. Furthermore, multiple linear regression analyses were conducted to investigate potential relationship between cumulative dust exposure and lung function parameters among the paper dust-exposed workers, adjusting for age, sex, and height. An interaction term between sex and cumulative dust exposure was included to investigate potential differences of this relationship between men and women. While age and cumulative dust showed a high correlation (r = 0.76, *p* < 0.001), we included age in this regression analysis because both variables influence lung function.

## Results

Out of 363 participants who attempted spirometry, 253 (69.7%) produced results that were both technically acceptable and valid. This included 160 individuals from the paper industry (exposed) and 93 from the water bottling industry (controls). The remaining 110 (30.3%) were excluded due to poor-quality or invalid spirometry maneuvers. In both groups the majority of workers were under the age of 35 years, however, the exposed group was older on average than the control group (Table 1). The average weight for the exposed and control groups was 60.2 kg and 57.1 kg, respectively, with corresponding BMIs of 22.8 and 21.2 kg/m^2^. The average number of hours worked per week was 48.1 in the exposed group and 50.7 in the control group. The average height of participants was 162 cm in the exposed group and 164 cm in the control group. None of the workers reported smoking.

**Table 1:** Personal characteristics of workers in the paper industry (exposed) and the water bottling industry (controls)

Variables	Categories	Exposed (*n* = 160)	Controls (*n* = 93)
Sex, *n* (%)	Male Female	102 (63.7) 58 (36.3)	51 (54.8) 42 (45.2)
Age (years), *n* (%)	18–25 years 26–34 years ≥ 35 years	55 (34.4) 51 (31.9) 54 (33.7)	53 (57.0) 31 (33.3) 9 (9.7)
Height (cm)	Mean (range)	162 (143–183)	164 (142–183)
Weight (kg)	Mean (range)	60.2 (38.0-102.0)	57.1 (41.0–88.0)
BMI (kg/m^2^)	Mean (range)	22.8 (15.4–32.8)	21.2 (16.3–32.9)
Hours per week	Mean (range)	48.1 (40.0–56.0)	50.7 (48.0–72.0)
Employment duration	Mean (range)	9.7 (1.0–45.0)	3.3 (1.0–15.0)
Educational Status, *n* (%)	Grade 1–12 Above grade 12	65 (40.6) 95 (59.4)	41 (44.1) 52 (55.9)
History of respiratory illness, *n* (%)	Yes No	5 (3.1) 155 (96.9)	3 (3.2) 90 (96.8)
Cooking food at home using biofuels, *n* (%)	Yes No	132 (83.0) 27 (17.0)	82 (88.2) 11 (11.8)
History of dust exposure in other industries, *n* (%)	Yes No	16 (10.0) 144 (90.0)	4 (4.3) 89 (95.7)

*n* = number of participants

The AM levels of inhalable paper dust collected were higher among female workers, measuring 5.0 mg/m^2^, compared to 4.2 mg/m^2^ in their male counterparts. In the preparation department, female workers were exposed to significantly higher levels of paper dust, averaging 8.8 mg/m^2^, while male workers had an average of 4.9 mg/m^2^ (Table 2). Female workers in the preparation section are primarily responsible for manually segregating or screening recycled paper from waste materials, while male workers typically operate the bell machine.

**Table 2:** Personal inhalable paper dust exposure (*n* = 150) by department and sex among 80 paper industry workers

Department	Male		Female	
	NW (NS)	Dust exposure (AM, range)	NW (NS)	Dust level (AM, range)
Preparation	9 (18)	4.9 (1.5–19)	11 (22)	8.8 (1.1–88.0)
Paper machine	18 (36)	2.8 (1.3–5.3)	2 (4)	2.5 (1.9–3.1)
Finishing and converting	12 (20)	5.6 (1.1–32)	8 (15)	2.5 (0.85–5.3)
Packing	7 (13)	4.8 (2.4–15)	13 (22)	3.4 (2.1–6.9)
Total	46 (87)	4.2 (1.1–32)	34 (63)	5.0 (0.85–88)

NW = Number of Workers, NS = Number of Samples, AM = Arithmetic Mean

Table 3 shows a significant difference in FEV_1_ between paper industry workers (2.62 l) and the control group (2.97 l) after adjusting for age, sex, and height. FVC and FEV_1_/FVC did not differ between the exposed and control groups.

When the analysis was stratified by sex, a significant difference in FEV_1_ between paper workers and controls was observed for females (Table 4). The mean FEV_1_ for female workers exposed to paper dust was 2.19 l compared to 2.84 l in the control group. Among males, the difference in FEV_1_ between exposed (2.86 l) and controls 3.09 l) was smaller and not statistically significant. There was no significant difference between exposed and controls for any other lung function parameters, for either males or females (Table 4).

**Table 3:** Lung function among workers exposed to paper dust (exposed) and water bottling workers (controls)

Lung function parameters	Exposed (*n* = 160)	Controls (*n* = 93)	
	AM, SD (range)	AM, SD (range)	*p* value
FEV_1_	2.62 ± 0.79 (0.97–4.62)	2.97 ± 0.69 (1.00–4.17)	0.002^a^
FVC	3.40 ± 1.03 (1.20–7.15)	3.47 ± 0.84 (2.17–5.96)	0.735^a^
FEV_1_/FVC	77.58 ± 10.7 (34.8–98.1)	78.6 ± 9.8 (48.6–96.3)	0.644^a^
FEV_1_/FVC < 70, *n* (%)	29 (18.1)	14 (15.1)	0.604^b^

^a^Covariance (ANCOVA) analysis to compare mean lung function parameters between groups, adjusting for age, sex and height, ^b^Pearson chi-Square analysis between exposed and controls, *n* = number of study participants, AM = Arithmetic mean, SD = Standard deviation, *p* value = significance level

**Table 4:** Lung function among workers exposed to paper dust (exposed) and water bottling workers (controls)

Lung function parameters	Females		*P* value	Males		*P* value
	Exposed (*n* = 58)	Controls (*n* = 42)		Exposed group (*n* = 102)	Controls (*n* = 51)	
	AM, SD (range)	AM, SD (range)		AM, SD (range)	AM, ± SD (range)	
FEV_1_	2.19 ± 0.66 (0.97–4.23)	2.84 ± 0.77 (1.00–3.99)	< 0.001^a^	2.86 ± 0.77 (0.97–4.62)	3.09 ± 0.61 (2.01–4.17)	0.860^a^
FVC	2.84 ± 0.83 (1.48–5.79)	2.94 ± 0.49 (2.17–4.14)	0.911^a^	3.73 ± 0.95 (1.20–7.15)	3.89 ± 0.84 (2.45–5.96)	0.677^a^
FEV_1_/FVC	77.7 ± 9.57 (48.0–98.1)	77.3 ± 8.3 (51.7–99.2)	0.200^a^	77.5 ± 11.4 (34.8–97.8)	79.7 ± 10.9 (48.6–96.3)	0.406^a^
FEV_1_/FVC < 70, *n* (%)	8 (13.8)	6 (14.3)	1.000^b^	21 (20.6)	8 (15.7)	0.519^b^

^a^Covariance (ANCOVA) analysis to compare mean lung function parameters between groups, stratified by sex, and adjusting for age and height, ^b^Fisher exact test between exposed and controls, *n* = number of study participants, AM = Arithmetic mean, SD = Standard deviation, *p* value = significance level

In the multiple linear regression analysis including only exposed workers, a significant interaction effect between sex and cumulative paper dust exposure was observed. Among male paper industry workers (*n* = 102), both FEV_1_ and FVC declined by 0.005 l for each unit increase in dust exposure (mg year/m^2^), after adjusting for age, height, and sex (Table 5). In female workers (*n* = 58), each unit increase in cumulative dust exposure was associated with a significant 0.010-liter decrease in both FEV_1_ and FVC.

**Table 5:** Association between cumulative dust exposure and lung function among 160 workers exposed to paper dust

Variables	Categories	FEV_1_			FVC			FEV_1_/FEVC		
		β	SE	*P* value	β	SE	*P* value	β	SE	*P* value
Intercept		−3.02	1.24	0.02	−3.23	1.62	0.05	63.90	19.98	0.02
Interaction: Sex (male = 1; female = 2). cumulative dust exposure	mg year/m^3^	−0.005	0.002	0.02	−0.005	0.003	0.04	−0.03	0.03	0.43
Sex	Male Female	Ref. −0.20	0.14	0.15	Ref. −0.33	0.18	0.07	Ref. 1.15	2.25	0.61
Age	18–25 years 26–34 years ≥ 35 years	Ref. −0.01 −0.21	0.13 0.17	0.93 0.22	Ref. 0.32 −0.07	0.17 0.22	0.07 0.75	Ref. −5.25 −4.43	2.12 2.67	0.01 0.09
Height	cm	0.04	0.01	< 0.001	0.04	0.01	< 0.001	0.11	0.12	0.37

Multiple linear regression of the association between cumulative dust exposure and lung function among workers exposed to paper dust, while adjusting for age, height, sex, and the interaction between sex and cumulative dust, β = unstandardized coefficients, SE = Standard Error, Ref. = Reference group.

**Fig. 1:**
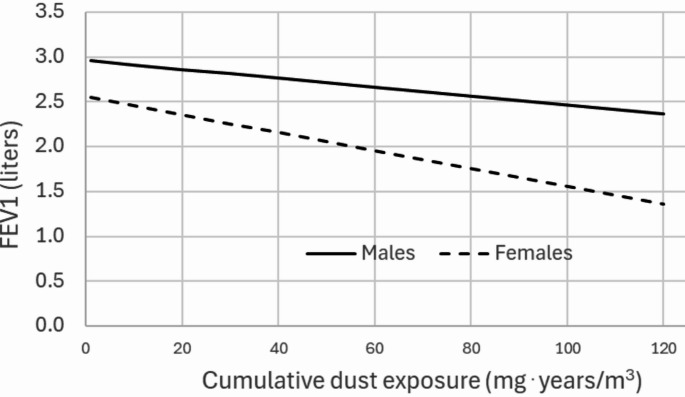
Association between cumulative dust exposure and forced expiratory volume in one second (FEV1) among male and female paper industry workers aged 26–34 years with a height of 162 cm

Based on the multiple linear regression, Fig. 1 illustrates the association between cumulative dust exposure and FEV_1_ for male and female paper industry workers in the age group 26–34 years with a height of 162 cm. The steeper slope observed among females indicates an interaction effect, suggesting a greater decline in FEV_1_ per unit of cumulative dust exposure compared to males. No significant association was observed between cumulative dust exposure and the FEV_1_/FVC ratio (Table 5).

## Discussion

The results showed a significant difference in lung function, as measured by FEV_1_, between paper industry workers and the control group after adjusting for age, sex, and height. When stratified by sex, the difference in FEV_1_ remained significant for females, but not among males. Furthermore, the results indicate a significant relationship between increasing cumulative paper dust exposure and reductions in FEV_1_ and FVC.

The forced expiratory volume in one second (FEV_1_) and forced vital capacity (FVC) decreased by 0.010 l among female workers and by 0.005 l among male workers for each unit increase in cumulative dust exposure. This finding indicated an exposure-response relationship between the amount of dust inhaled over time (measured in mg year/m^2^) and lung function impairment after adjusting for sex, age, and height. The findings suggest that female workers experience more pronounced airflow reductions than male workers, due to paper dust exposure in the workplace. For instance, if workers are exposed to 5 mg/m^3^ over 10 years, the cumulative exposure will be 50 mg year/m^3^. This level of exposure is associated with an additional decline in FEV_1_ of 0.5 l in women and 0.25 l in men over the 10-year period. These finding are compatible with the reduced FEV_1_ observed among paper workers with an average employment duration of 9.7 years compared to controls showing a difference of 0.65 l among females and 0.23 l among males.

Notably, no significant differences in lung function were observed among male employees when comparing those exposed to paper dust and the control group. However, the observed difference in findings when comparing the sexes may partially be explained by differing exposure levels, with female workers experiencing higher average exposures (5.0 mg/m^2^) compared to males (4.2 mg/m^2^). Female workers in the preparation area, who manually sorted recycled paper from waste, had the highest dust exposure in the factories, averaging 8.8 mg/m^2^.

The findings of this study align with previous research published from both the Swedish (Andersson et al. 2020) and German (Kraus et al. 2004) soft tissue paper industries, both of which reported an association between paper dust exposure and reduced lung function (Andersson et al. 2020; Kraus et al. 2004). The German study, published in 2004, and the Swedish study, published in 2020, employed methodologies similar to that of the present study. However, both studies included smokers and therefore needed to adjust for smoking in their analyses. The German study reported higher exposure levels than those observed in our present study, while the Swedish study reported similar exposure levels. Also, our findings are consistent with a study conducted in the Croatian paper recycling sector (Zuskin et al. 1998), further supporting the association between paper dust exposure and reduced lung function.

On the other hand, another Swedish study, from a different soft tissue paper mill, reported no significant differences in lung function between paper dust-exposed workers and a control group (Thorén et al. 1989). However, this study was from 1989, and the dust exposure levels were lower than in our present study.

A notable strength of this study is that all the workers were non-smokers. Smoking is widely recognized as a significant risk factor that can lead to reduced lung function, and it has been a challenge in all previous studies of this topic to adjust for this factor. With only non-smokers in the population, the results are clearly more valid. Another strength of the study is the use of objective, personally worn dust measurements collected in the participating factories. Such measurement data are rare for industrial settings on the African continent, and no prior data of this kind were available for comparison. As a result, cumulative dust exposure levels were calculated based on estimated dust levels in the different factory departments. We developed a job exposure matrix (JEM) to quantify cumulative dust exposure for each worker, considering the variations in exposure across departments within and between the four industries, as most workers occupied multiple positions throughout their tenure in the paper industry, thus falling into more than one exposure category. In estimating cumulative exposure, males and females were assigned the same exposure levels if they had worked in the same departments. This approach was taken due to an insufficient number of dust measurements within each department to allow for reliable stratification by sex. Therefore, cumulative dust was not calculated for female workers separately, although this would have been valuable given indications from the measurements that females may have experienced different exposure levels than males. Future studies should aim to investigate sex-specific differences in exposure more thoroughly.

It is also a notable strength of the study to use spirometry to assess lung function, using ATS guidelines, and to compare the dust-exposed workers with a control group of water-bottling workers with a low dust exposure.

A bronchodilator test was not included in this study, as our focus was on detecting early signs of occupational lung impairment caused by dust exposure rather than diagnosing airway obstruction. Moreover, baseline spirometry provided sufficient data for evaluating lung function decline linked to cumulative dust exposure.

This study does have some weaknesses. The design was cross-sectional, reducing the possibility of demonstrating any causality between exposure and lung function outcomes. This challenge was reduced by using regression analyses to study the exposure-response relationship in the study. However, in the study, we presumed that dust exposure levels remained constant within job groups while estimating cumulative exposure. This might not be correct and may have introduced a bias. Therefore, we recommend that future studies of paper dust exposure and respiratory health adopt a longitudinal approach. Another weakness is that the investigation did not account for any chemicals involved in the paper manufacturing process, such as those found in recycled paper, including glues, resins, xylene, and binding agents, all of which might be present as volatile organic compounds in the production areas of the included factories. Bacteria and fungi may also be present in this work environment but were not studied in the present investigation.

Also, the cross-sectional design may have introduced a healthy worker effect, as individuals with chronic respiratory impairment may have left the workplace prior to data collection, potentially leading to an underestimation of the true impact of dust exposure on lung function. However, despite the limitations of the cross-sectional design, a significant difference in lung function variables was observed between exposed workers and the control group. Some invalid spirometry results were excluded from the data analysis. This is due to factors such as insufficient effort or incomplete exhalation, hesitation at the onset, premature termination of the breath, and fatigue might impair a worker’s ability to maintain consistent performance. Future studies may benefit from studying larger populations, encompassing both male and female workers. A greater sample size would enable stratification by age groups, allowing for more precise control of age-related effects, and potentially offering a more robust alternative to the regression-based adjustments used in the present study.

## Conclusions

This findings indicate that cumulative paper dust exposure is associated with a decline in lung function variables, being more pronounced among female workers that among males. Based on these results, we recommend that the paper industry implement effective dust control strategies to alleviate the detrimental effects of paper dust exposure on workers’ lung function.

## Data Availability

The raw data supporting the conclusions of this article will be made available by the authors on request.
